# Supplementary material for: Checkpoint kinase 1/2 inhibition potentiates anti-tumoral immune response and sensitizes gliomas to immune checkpoint blockade
Source: Nat Commun. 2023 Mar 22;14:1566. doi: 10.1038/s41467-023-36878-2 (PMC10033639; doi:10.1038/s41467-023-36878-2)

Checkpoint kinase 1/2 inhibition potentiates anti-tumoral immune response and sensitizes gliomas to immune checkpoint blockade

Crismita Dmello[1,2,#,*], Junfei Zhao[3,4,#], Li Chen[1,2], Andrew Gould[1,2], Brandyn Castro,[1,5], Victor A Arrieta[1,2,6], Daniel Y. Zhang[1,2], Kwang-Soo Kim[1,2], Deepak Kanojia[1,2], Peng Zhang[1,2], Jason Miska[1,2], Ragini Yeeravalli[1,2], Karl Habashy[1,2], Ruth Saganty[1,2], Seong Jae Kang[1,2], Jawad Fares[1,2], Connor Liu[7,8], Gavin Dunn[7,8,9], Elizabeth Bartom[10], Matthew J Schipma[11], Patrick D. Hsu[12,13,14], Mahmoud S Alghamri[15,16], Maciej S. Lesniak[1,2], Amy B. Heimberger[1,2], Raul Rabadan[3,4,17], Catalina Lee-Chang[1,2,*], Adam M. Sonabend[1,2,*]

[1]Department of Neurological Surgery, Feinberg School of Medicine, Northwestern University, Chicago, IL, USA

[2]Northwestern Medicine Malnati Brain Tumor Institute of the Lurie Comprehensive Cancer Center, Feinberg School of Medicine, Northwestern University, Chicago, IL, USA

[3]Program for Mathematical Genomics, Department of Systems Biology, Columbia University, New York, NY, USA

[4]Department of Biomedical Informatics, Columbia University, New York, NY, USA

[5]Section of Neurological Surgery, University of Chicago Medicine, Chicago, IL, USA

[6]PECEM, Facultad de Medicina, Universidad Nacional Autónoma de México, Mexico City, Mexico

[7]Department of Neurological Surgery, Washington University School of Medicine, St Louis, MO, USA

[8]Department of Pathology and Immunology, Washington University School of Medicine, St Louis, MO, USA

[9]The Alvin J. Siteman Cancer Center at Barnes-Jewish Hospital and Washington University School of Medicine, St Louis, MO, USA

[10]Department of Biochemistry and Molecular Genetics, Northwestern University, Chicago, IL, USA

[11]NUSeq Core, Center for Genetic Medicine, Feinberg School of Medicine, Northwestern University, Chicago, IL, USA

[12]Innovative Genomics Institute, University of California, Berkeley, Berkeley, CA, USA

[13]Department of Bioengineering, University of California, Berkeley, Berkeley, CA, USA

[14]Center for Computational Biology, University of California, Berkeley, Berkeley, CA, USA

[15]Department of Neurosurgery, University of Michigan Medical School, Ann Arbor, MI, USA

[16]Department of Cell and Developmental Biology, University of Michigan Medical School, Ann Arbor, MI, USA

[17]Department of Neurology, Department of Pathology, Institute for Cancer Genetics, Columbia University Medical Center, New York, NY, USA

[#]These authors contributed equally: Crismita Dmello, Junfei Zhao.

[*]These authors jointly supervised this work: Crismita Dmello, Catalina Lee-Chang, Adam M. Sonabend. e-mail: crismitadmello@northwestern.edu; catalina.leechang@northwestern.edu; adam.sonabend@nm.org

Supplementary Table 1. Bar code details for the amplification of the sgRNAs for CRISPR screen 1 and 2. The P5 forward primer mix was used as a forward primer for both the CRISPR screens.

Name of the sample	Barcodes	Sequence
**CRISPR screen 1**		
Library	SEQ4	CAAGCAGAAGACGGCATACGAGATATTCTAGGGTGACTGGAGTTCAGACGTGTGCTCTTCCGATCTCCGACTCGGTGCCACTTTTTCAA
Day 0	SEQ8	CAAGCAGAAGACGGCATACGAGATTTGAATAGGTGACTGGAGTTCAGACGTGTGCTCTTCCGATCTCCGACTCGGTGCCACTTTTTCAA
WT	SEQ5	CAAGCAGAAGACGGCATACGAGATCGTTACCAGTGACTGGAGTTCAGACGTGTGCTCTTCCGATCTCCGACTCGGTGCCACTTTTTCAA
CD8 KO	SEQ7	CAAGCAGAAGACGGCATACGAGATTTACGCACGTGACTGGAGTTCAGACGTGTGCTCTTCCGATCTCCGACTCGGTGCCACTTTTTCAA
**CRISPR screen 2**		
Library	SEQ3	CAAGCAGAAGACGGCATACGAGATGAAGAAGTGTGACTGGAGTTCAGACGTGTGCTCTTCCGATCTCCGACTCGGTGCCACTTTTTCAA
Day 0	SEQ8	CAAGCAGAAGACGGCATACGAGATTTGAATAGGTGACTGGAGTTCAGACGTGTGCTCTTCCGATCTCCGACTCGGTGCCACTTTTTCAA
WT D18-D23	SEQ6	CAAGCAGAAGACGGCATACGAGATGTCTGATGGTGACTGGAGTTCAGACGTGTGCTCTTCCGATCTCCGACTCGGTGCCACTTTTTCAA
WT D24-D38	SEQ7	CAAGCAGAAGACGGCATACGAGATTTACGCACGTGACTGGAGTTCAGACGTGTGCTCTTCCGATCTCCGACTCGGTGCCACTTTTTCAA
CD8 KO D18-D23	SEQ4	CAAGCAGAAGACGGCATACGAGATATTCTAGGGTGACTGGAGTTCAGACGTGTGCTCTTCCGATCTCCGACTCGGTGCCACTTTTTCAA
CD8 KO D24-D38	SEQ5	CAAGCAGAAGACGGCATACGAGATCGTTACCAGTGACTGGAGTTCAGACGTGTGCTCTTCCGATCTCCGACTCGGTGCCACTTTTTCAA

**P5- Forward Primer mix (mixture of 8 primers)**
P5 0 nt stagger AATGATACGGCGACCACCGAGATCTACACTCTTTCCCTACACGACG
CTCTTCCGATCTTTGTGGAAAGGACGAAACACCG
P5 1nt stagger AATGATACGGCGACCACCGAGATCTACACTCTTTCCCTACACGACG
CTCTTCCGATCTCTTGTGGAAAGGACGAAACACCG
P5 2 nt stagger AATGATACGGCGACCACCGAGATCTACACTCTTTCCCTACACGACG
CTCTTCCGATCTGCTTGTGGAAAGGACGAAACACCG
P5 3 nt stagger AATGATACGGCGACCACCGAGATCTACACTCTTTCCCTACACGACG
CTCTTCCGATCTAGCTTGTGGAAAGGACGAAACACCG
P5 4 nt stagger AATGATACGGCGACCACCGAGATCTACACTCTTTCCCTACACGACG
CTCTTCCGATCTCAACTTGTGGAAAGGACGAAACACCG
P5 6 nt stagger AATGATACGGCGACCACCGAGATCTACACTCTTTCCCTACACGACG
CTCTTCCGATCTTGCACCTTGTGGAAAGGACGAAACACCG
P5 7nt stagger AATGATACGGCGACCACCGAGATCTACACTCTTTCCCTACACGACG
CTCTTCCGATCTACGCAACTTGTGGAAAGGACGAAACACCG
P5 8nt stagger AATGATACGGCGACCACCGAGATCTACACTCTTTCCCTACACGACG
CTCTTCCGATCTGAAGACCCTTGTGGAAAGGACGAAACACCG

Supplementary Table 2. Mouse specific primer sequences for **a,** quantitative real time PCR and **b,** single gene CRISPR Cas9 Knockout.

**a**	**Single gene KO sgRNA sequence (Mouse specific)**	
**Gene**	**5'-3'**	**Length**
NTC F	CACCAATATTTGGCTCGGCTGCGC	24
NTC R	AAACGCGCAGCCGAGCCAAATATT	24
Chek2 F	CACCGGCTGGAGACAGTGTCTACCC	25
Chek2 R	AAACGGGTAGACACTGTCTCCAGCC	25

**b**	**Quantitative real time PCR primers (Mouse specific)**	
**Gene**	**5'-3'**	**Length**
IFNβ F	TCCGAGCAGAGATCTTCAGGAA	22
IFNβ R	TGCAACCACCACTCATTCTGAG	22
ISG15 F	GGAACGAAAGGGGCCACAGCA	21
ISG15 R	CCTCCATGGGCCTTCCCTCGA	21
IRF7 F	ATGCACAGATCTTCAAGGCCTGGGC	25
IRF7 R	GTGCTGTGGAGTGCACAGCGGAAGT	25
IFNα F	GGACTTTGGATTCCCGCAGGAGAAG	26
IFNα R	GCTGCATCAGACAGCCTTGCAGGTC	25
PD-L1 F	ATTGCTCCTTGACTGCTGGCTG	22
PD-L1 R	TTCTGGGTTCCTCCTCCTTTCC	22
GAPDH F	CATCACTGCCACCCAGAAGACTG	23
GAPDH R	ATGCCAGTGAGCTTCCCGTTCAG	23

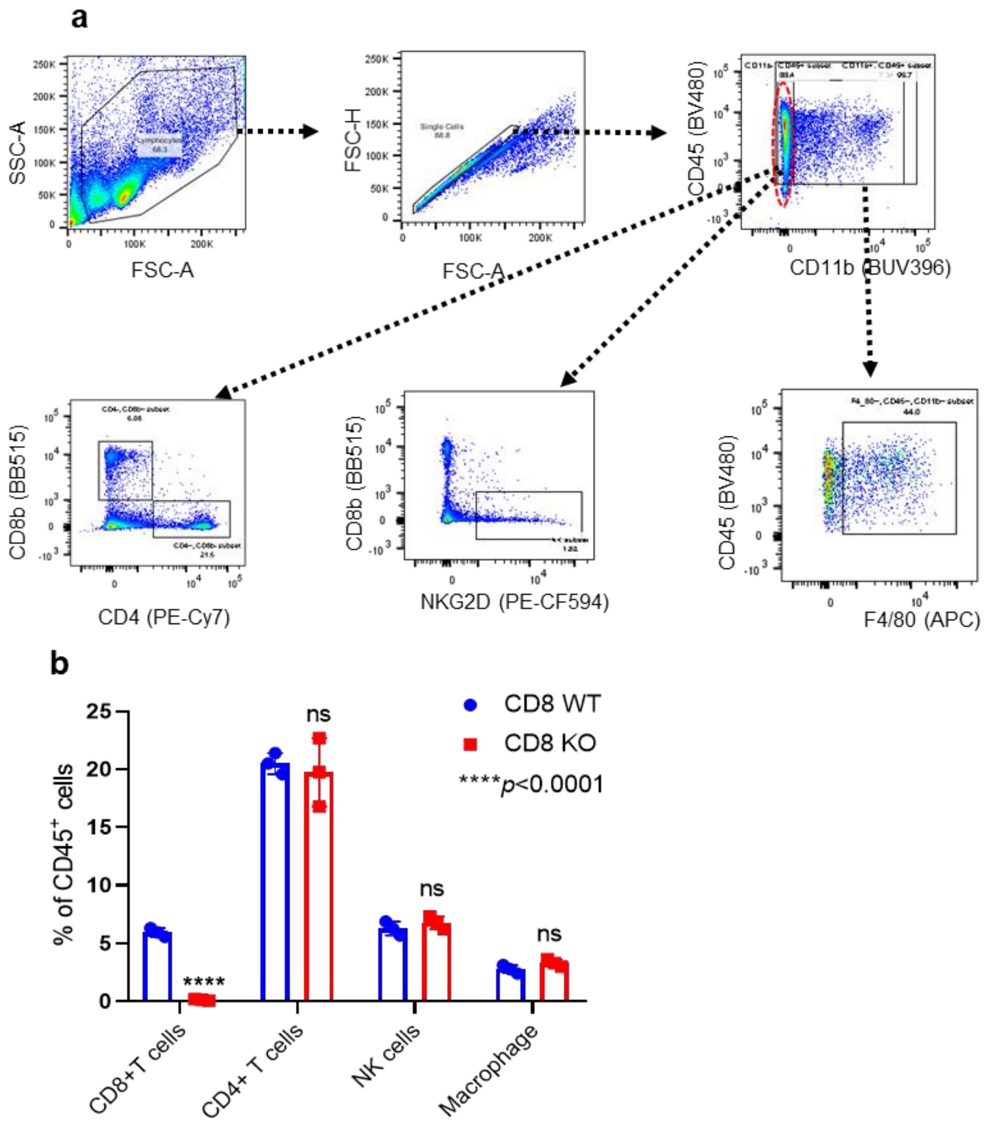

Supplementary Figure 1. Immune profiling of CD8 KO mice. a, Representative density plots from flow cytometry analysis of immune cells from the spleen of the WT mouse. **b,** Graphical representation of percentages of different immune populations in the spleen of WT and CD8 KO mice using flow cytometry (n=3 mice/group). The error bars represents mean ± SD. The statistics was performed using Sidak's multiple comparisons test.

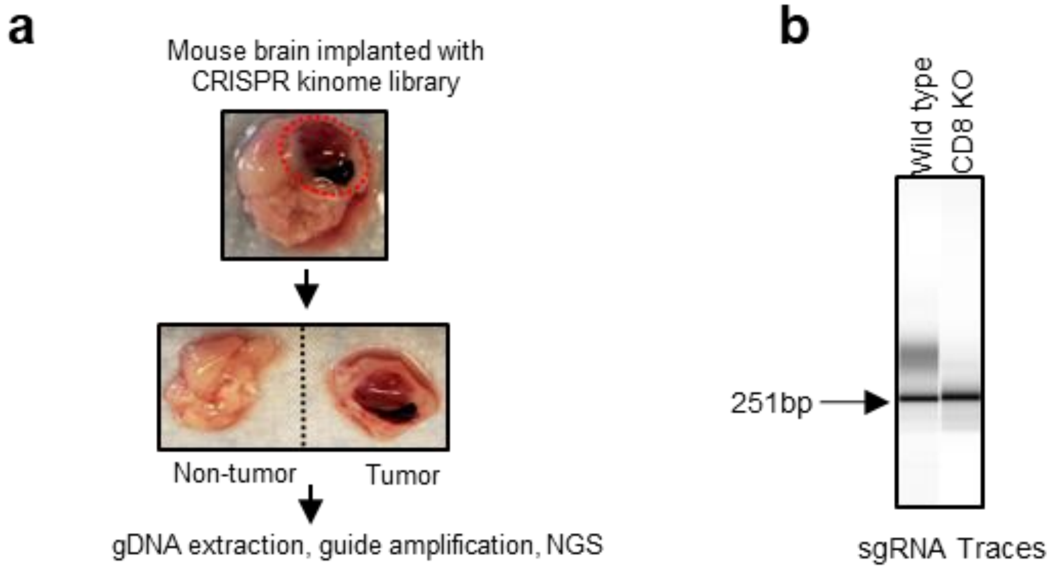

Supplementary Figure 2. *In-vivo* CRISPR screen guide extraction and counts. a, Representative image of a mouse brain cut into tumor and non-tumor region. The tumor region was used for genomic DNA extraction and subsequent sgRNA amplification. **b,** Agarose gel showing guide purity for each of the samples: pooled guides from wild type mice (n=11 mice) and pooled guides from CD8 KO mice (n=9 mice).

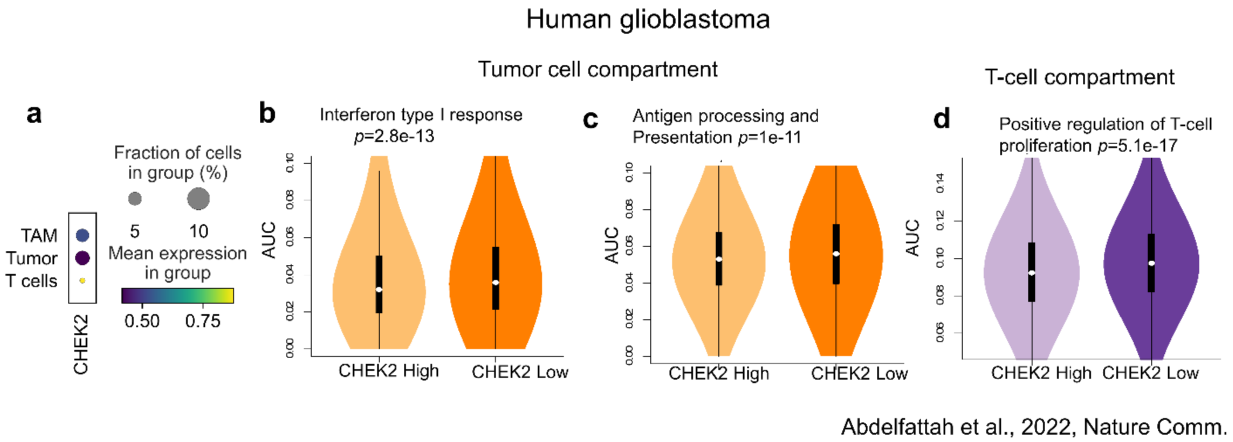

Supplementary Figure 3. *CHEK2* expression in tumor cells is inversely associated with Interferon type I response and enhanced antigen presentation on tumor cells, in human GBM patients. a, The figure showing the expression of *CHEK2* in tumor associated macrophages (TAM), tumor cells and T cells. The violin plots of the gene signature scores of **b,** interferon type I response and **c,** antigen processing and presentation pathway in n=57,534 tumor cells with high and low *CHEK2* expression. The violin plots of the gene signature scores of **d,** T-cell proliferation pathway in n=7,767 T cells from high *CHEK2* and low *CHEK2* expressing samples. The scRNA-seq data was used from the study published by Abdelfattah et al., 2022[1]. For (b-d), the p-value represents two-tailed Mann-Whitney test; whiskers represent minimum and maximum values, the white dot inside the box represents the median and the box extends from the 25th to 75th percentiles.

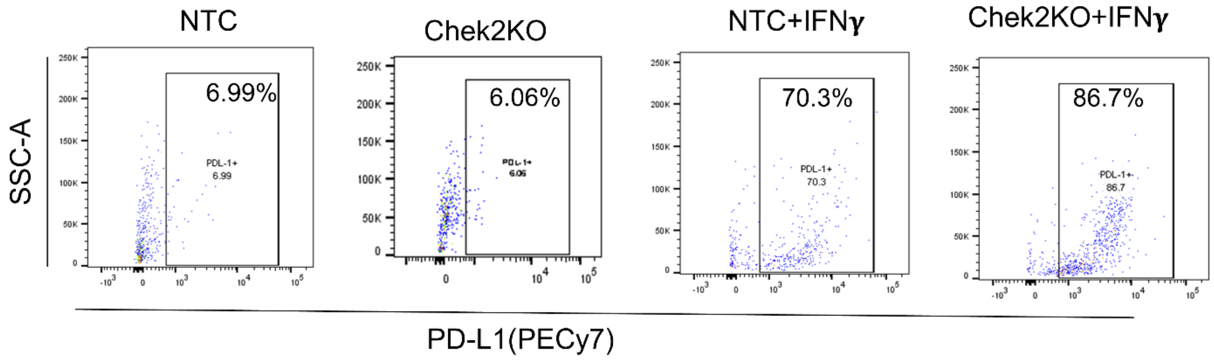

Supplementary Figure 4. Representative flow cytometry plots showing surface expression of PDL1 on GL261 Chek2 KO and NTC clones, at the basal level and upon stimulation with IFNγ for 48 h, presented on Fig. 3g and Fig. 5b.

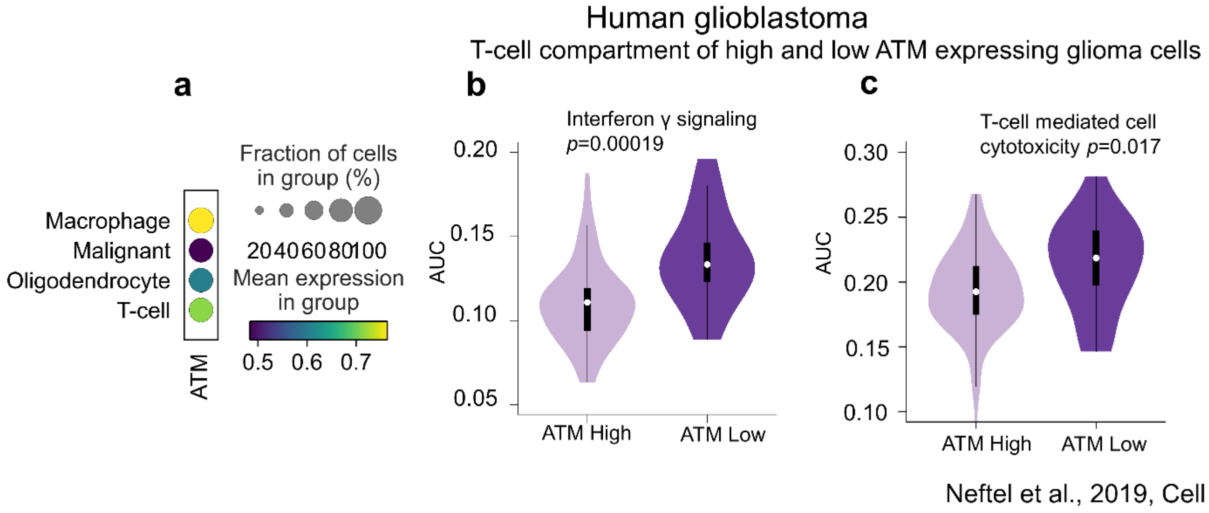

Supplementary Figure 5. *ATM* expression in tumor cells is inversely associated with enhanced antigen presentation on tumor cells, in human GBM patients. a, Figure showing the expression of *ATM* in macrophages, tumor cells, oligodendrocytes and T cells. The violin plots of the gene signature scores of **b,** Interferon γ signaling and **c,** T-cell mediated cytotoxicity pathway in n=94 T cells from high *ATM* vs low *ATM* expressing samples. T cells were analyzed from the Neftel et al., 2019 scRNA-seq dataset[2]. For (b-c), the p-value represents two-tailed Mann-Whitney test; whiskers represent minimum and maximum values, the white dot inside the box represents the median and the box extends from the 25th to 75th percentiles.

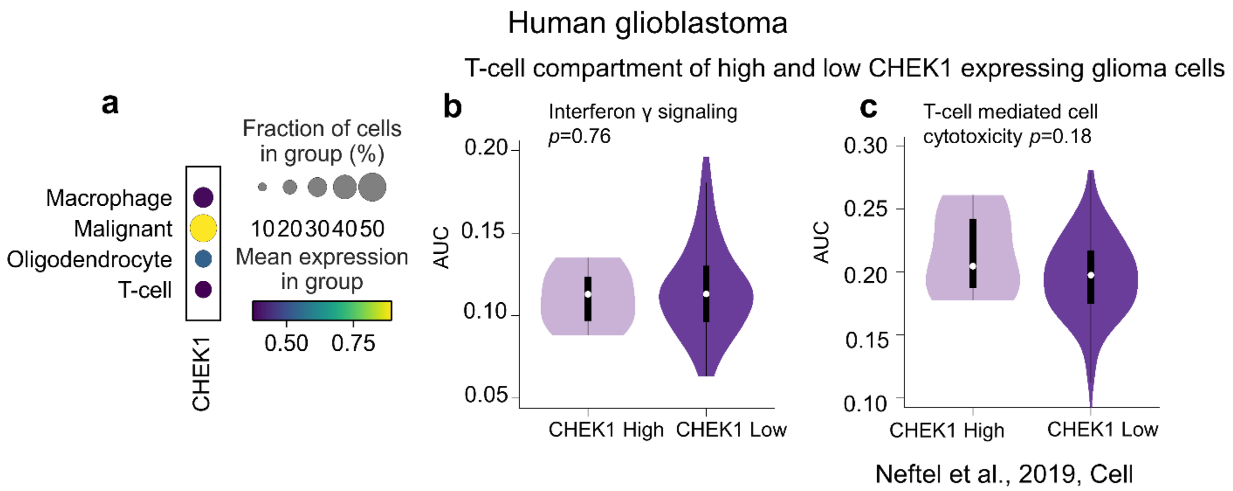

Supplementary Figure 6. ***CHEK1*** **expression in tumor cells does not correlate with T-cell phenotype in human GBM patients. a,** Figure showing the expression of *CHEK1* in macrophages, tumor cells, oligodendrocytes and T cells. The violin plots of the gene signature scores of **b,** Interferon γ signaling and **c,** T-cell mediated cytotoxicity pathway in n=94 T cells from high *CHEK1* vs low *CHEK1* expressing samples. T cells were analyzed from the Neftel et al., 2019 scRNA-seq dataset[2]. For (b-c), the p-value represents two-tailed Mann-Whitney test; whiskers represent minimum and maximum values, the white dot inside the box represents the median and the box extends from the 25th to 75th percentiles.

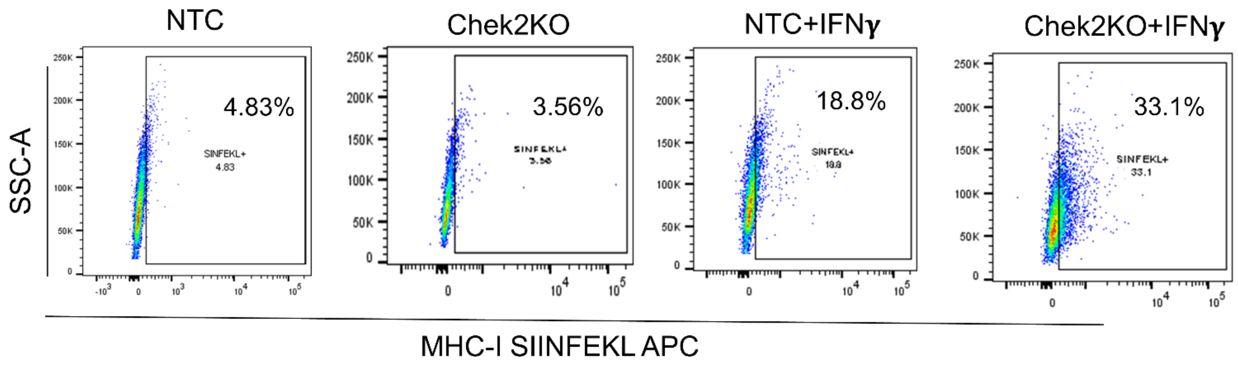

Supplementary Figure 7. Representative flow cytometry plots showing surface expression of MHC-I SIINFEKL on GL261 Chek2 KO and NTC clones, at the basal level and upon stimulation with IFNγ for 48 h, presented on Fig. 4d.

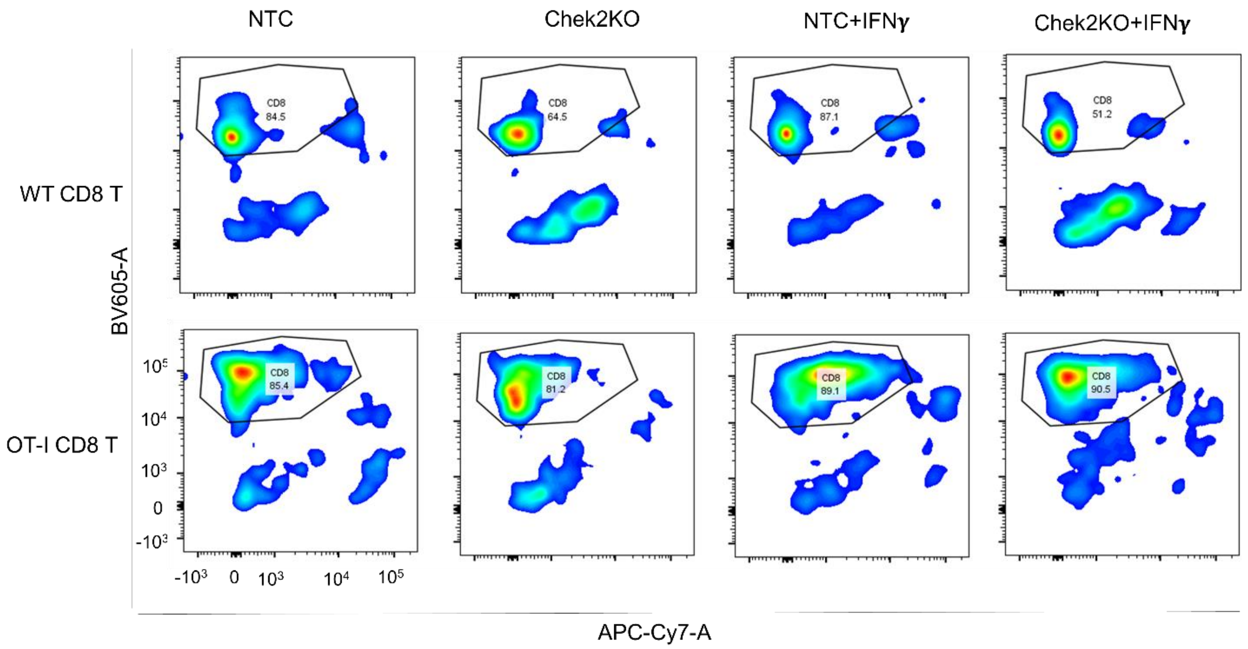

Supplementary Figure 8. Gating strategy for WT and OT-1 CD8 T cells co-cultured with Chek2 KO and control cells, presented on Fig. 4e.

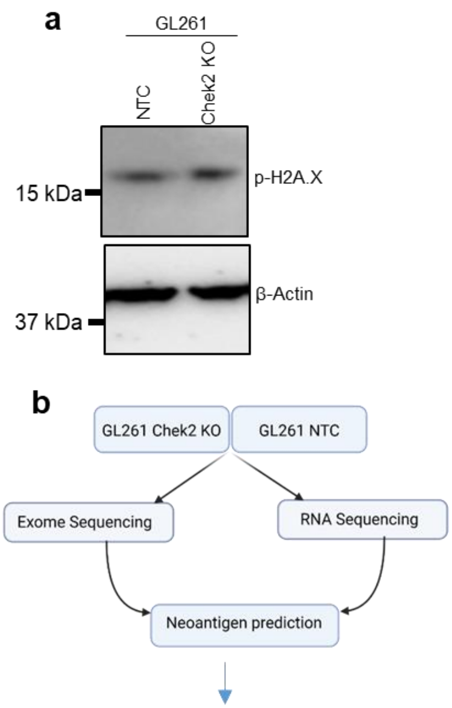

c

GL261 Chek2 KO vs. GL261 NTC

Gene Name	Mutant Epitope Seq	Gene Expression	Variant Type	Mutation	Median mutant score
Chek2	VSPRNSVLF	21.227	FS	T/X	379.957
Olfr77	THINNIFLYM	0.199	missense	L/M	878.83
Laptm4b	VWYLTYAL	53.06	missense	C/Y	72.567
Dytn	LSWAQCEPL	0.039	missense	S/C	691.03

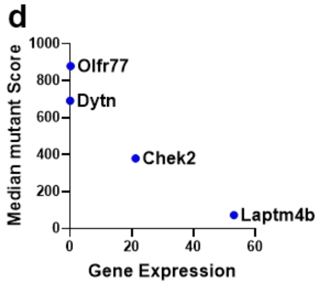

pVAC-seq pipeline (http://www.pvactools. org/)

Supplementary Figure 9. Chek2 depletion neither increases DNA damage nor neoantigen predictions in glioma cells. a, Baseline phosphorylation of γH2A.X in GL261 non-targeting control (NTC) and Chek2 KO cells. N=2 independent replicates. **b,** Schematic representation of the workflow to study neonantigen predictions in GL261 NTC and Chek2 KO cells. **c,** Neonantigen prediction analysis comparing Chek2 knockout with NTC. Table showing the genes with median mutant score <1000 for neoantigens that are unique to GL261 Chek2 KO and are absent in GL261 NTC. **d,** Graphical representation of gene expression versus neoantigens with median mutant score <1000, unique to GL261 Chek2 KO as compared to GL261 NTC cells.

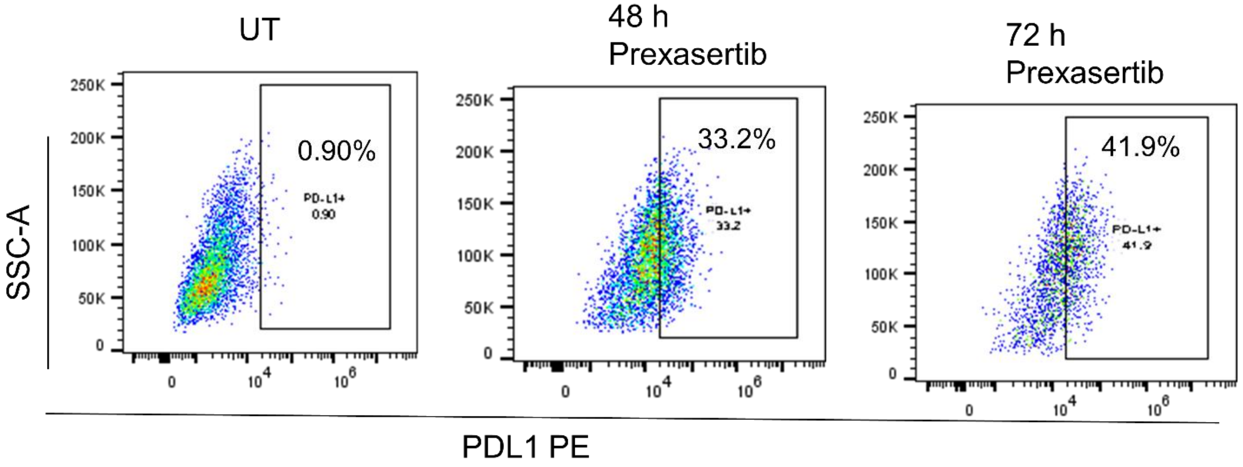

Supplementary Figure 10. Representative flow cytometry plots showing surface expression of PD-L1 on GL261 glioma cells treated with Prexasertib (300nmol/L) at the indicated time points, presented on Fig. 5c.

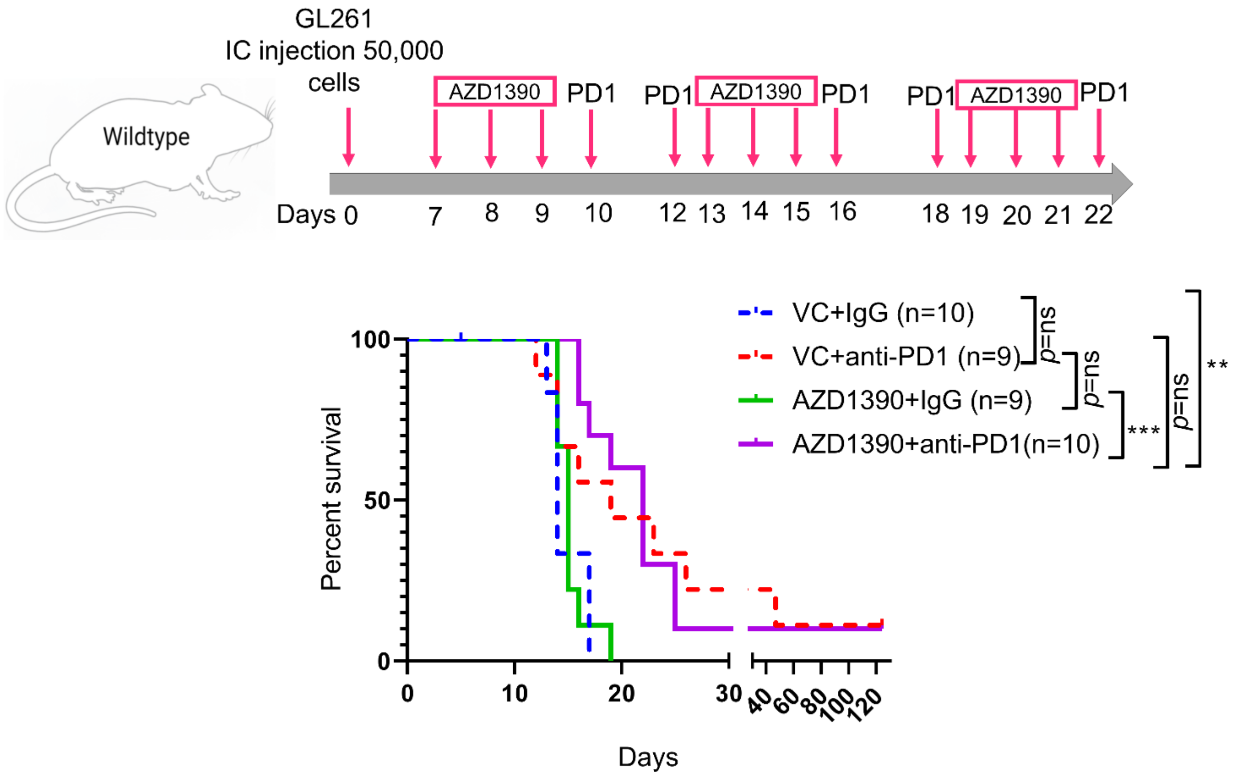

Supplementary Figure 11: Combination of ATM inhibitor (AZD1390) and PD-1 blockade improves survival in glioma-bearing mice. The schematic representation of the dosing scheme for AZD1390 and anti-PD-1. KM survival curves for C57BL/6 mice bearing GL261 glioma. 7 days after intracranial tumor implantation, the animals were randomized into 4 groups: vehicle and isotype control (IgG), anti-PD-1, AZD1390 (ATM inhibitor), and AZD1390 and the anti-PD-1 combination group. Survival analysis was performed using the log-rank test. The median survival duration in the treatment groups were as follows: VC + IgG, 14 days; VC + anti-PD-1, 19; AZD1390 + IgG, 15 days; AZD1390 + anti-PD-1, 22 days. Statistics: VC + IgG vs VC+ anti-PD-1, p=0.07; VC + IgG vs AZD1390 + IgG, p=0.6; and VC + IgG vs AZD1390 + anti-PD-1, p=0.002.

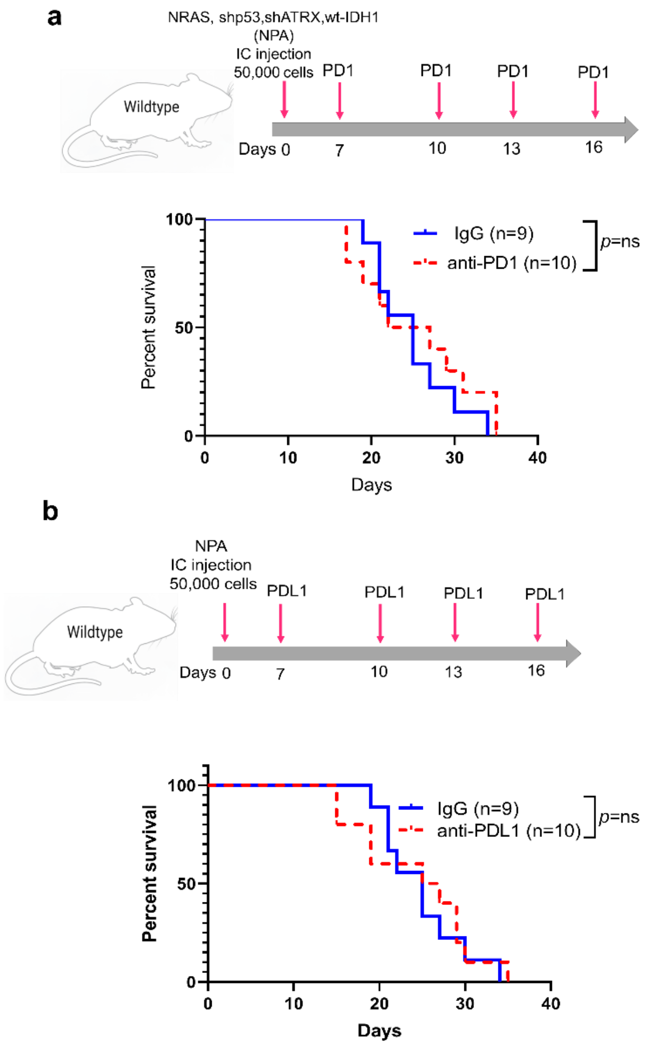

Supplementary Figure 12. The NPA glioma model is non-responsive to PD-1 and PD-L1 blockade. . a, The schematic representation of the dosing scheme followed for the survival experiment. KM survival curves of the C57BL/6 mice bearing NPA glioma. One group of mice was treated with an anti-PD-1 antibody (n=10) and another group of mice was treated with the isotype control antibody (n=9). 50,000 NPA neurospheres cells were implanted/mouse. Survival analysis was performed using the log-rank test. The median survival duration in the treatment groups were as follows: IgG, 25 days; anti-PD-1, 24.5 days; Statistics: anti-PD-1 vs IgG, p=0.49. **b,** The schematic representation of the dosing scheme followed for the survival experiment. KM survival curves of the C57BL/6 mice bearing NPA glioma. One group of mice was treated with an anti-PDL1 antibody (n=10) and another group of mice was treated with the isotype control antibody (n=9). 50,000 NPA neurospheres cells were implanted/mouse. Survival analysis was performed using the log-rank test. The median survival duration in the treatment groups were as follows: IgG, 25 days; anti-PDL1, 26 days; Statistics: anti-PD-L1 vs IgG, p=0.78.

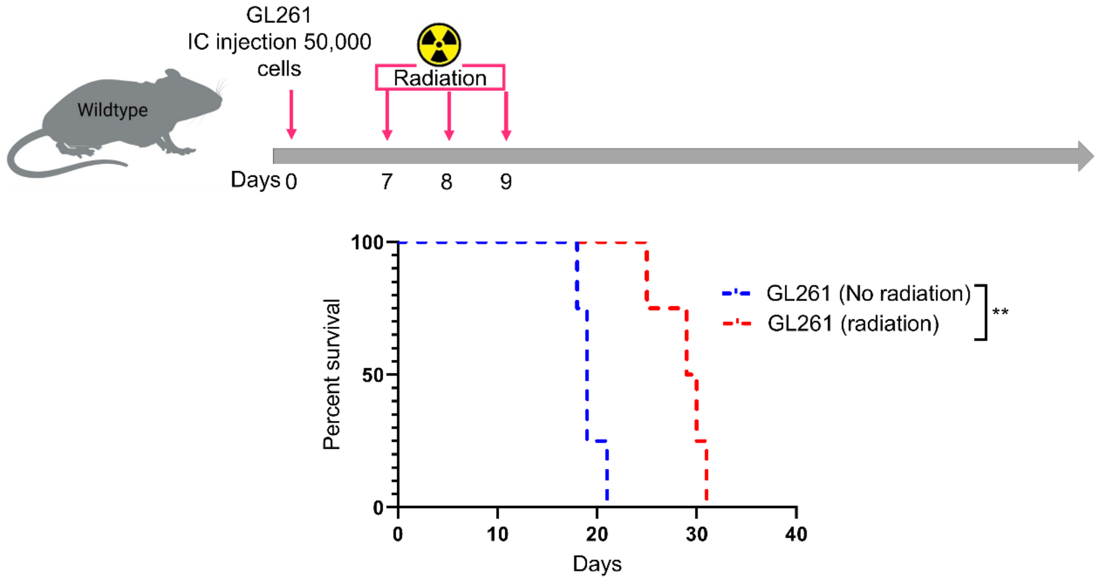

Supplementary Figure 13. Radiotherapy extends the survival of GL261 glioma bearing mice by 10 days. KM survival curves for GL261 glioma bearing C57BL/6 mice. On the 7th day post injection, the animals were randomized into 2 groups (4 animals/group) and one group of animals was irradiated with 3 Gy radiation for 3 consecutive days and monitored for survival study. Survival analysis was performed using the log-rank test. The MS durations in the treatment groups were as follows: No radiation, 19 days; Radiation, 29.5 days; Statistics: Radiation versus No radiation, p=0.006.

References

1. Abdelfattah, N., *et al.* Single-cell analysis of human glioma and immune cells identifies S100A4 as an immunotherapy target. *Nat Commun* **13**, 767 (2022).
2. Neftel, C., *et al.* An Integrative Model of Cellular States, Plasticity, and Genetics for Glioblastoma. *Cell* **178**, 835-849 e821 (2019).

Supplementary Figure 14. Source data of the supplementary Figures.

Source data of Supplementary Figure 2

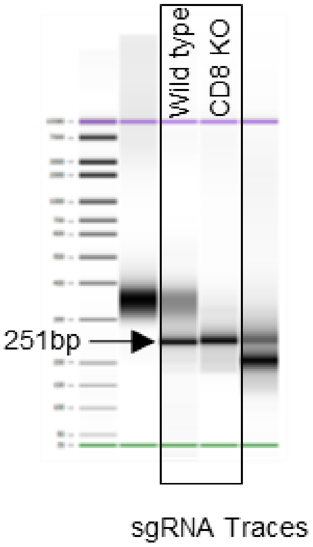

Source data of Supplementary Figure 3

The graphs were made using the Abdelfattah et al.[1] scRNA-seq publicly available data GEO #GSE182109 (https://www.ncbi.nlm.nih.gov/geo/query/acc.cgi?acc=GSE182109).

Source data of Supplementary Figure 5 and 6

The graphs were made using the Neftel et al.[2] scRNA-seq publicly available data GEO #GSE131928 (https://www.ncbi.nlm.nih.gov/geo/query/acc.cgi?acc=GSE131928).

Source data of Supplementary Figure 9a

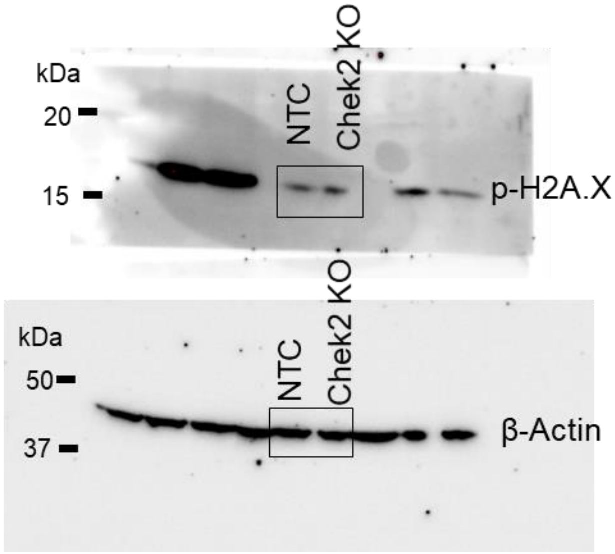

Supplement: Supplementary file 1 — Supplementary Information [file 41467_2023_36878_MOESM1_ESM.pdf]
