# Supplementary material for: Checkpoint kinase 1/2 inhibition potentiates anti-tumoral immune response and sensitizes gliomas to immune checkpoint blockade
Source: Nat Commun. 2023 Mar 22;14:1566. doi: 10.1038/s41467-023-36878-2 (PMC10033639; doi:10.1038/s41467-023-36878-2)
Supplement: Supplementary file 3 — Description of Additional Supplementary Files [file 41467_2023_36878_MOESM3_ESM.pdf]

Description of Additional Supplementary Files

Supplementary Data 1: List of gene sets.
The list contains gene ontology term enriched gene sets for the data presented on figures 3d-e, 4b, supplementary figures 3b-d, 5b-c, 6b-c.
